# Relationship between serum and tear levels of tissue plasminogen activator and plasminogen activator inhibitor-1 in diabetic retinopathy

**DOI:** 10.1186/s12886-022-02550-4

**Published:** 2022-09-03

**Authors:** Nurbadriah binti Jasmiad, Rohana binti Abd Ghani, Renu Agarwal, Zaliha binti Ismail, Azlindarita Aisyah Mohd Abdullah, Mohd Yusri Idorus

**Affiliations:** 1 Department of Internal Medicine, Faculty of Medicine, Universiti Teknologi MARA (UiTM), Sungai Buloh, Selangor Darul Ehsan Malaysia; 2 Institute of Pathology, Laboratory and Forensic Medicine (I-PPerForM), Universiti Teknologi MARA (UiTM), Sungai Buloh, Selangor Darul Ehsan Malaysia; 3 Department of Pharmacology and Therapeutics, School of Medicine, International Medical University, Kuala Lumpur, Malaysia; 4 Department of Public Health Medicine, Faculty of Medicine, Universiti Teknology MARA (UiTM), Sungai Buloh, Selangor Darul Ehsan Malaysia; 5 MSU Medical Centre, Persiaran Olahraga, Shah Alam, Selangor Darul Ehsan Malaysia; 6 MSU Clinical Centre of Excellence, Management and Science University, Shah Alam, Selangor Darul Ehsan Malaysia; 7 Institute of Medical Molecular Biotechnology (IMMB), Faculty of Medicine, Universiti Teknologi MARA (UiTM), Sungai Buloh, Selangor Darul Ehsan Malaysia

**Keywords:** Type 2 diabetes mellitus, Diabetic retinopathy, Tissue plasminogen activator (tPA), Plasminogen activator inhibitor-1 (PAI-1)

## Abstract

**Background:**

Diabetic retinopathy (DR) is a serious complication of longstanding type 2 diabetes mellitus (T2DM), a leading cause of blindness and visual disability in the world. The aim of this study is to compare the activity of tissue plasminogen activator (tPA) and plasminogen activator inhibitor-1 (PAI-1) in tears and serum of patients with DR and those without DR.

**Method:**

Among the T2DM patients enrolled in this study, 26 patients had DR (*n* = 26) while 29 were without DR (*n* = 29). The blood and tear samples were obtained from all participants. The level of PAI-1 and tPA were measured in both the serum and tears. Anthropometric measurements, HbA1c, renal and lipid profile were also obtained.

**Results:**

Patients with DR had significantly longer disease duration and higher systolic blood pressure compared to those without DR. Serum PAI-1 level was significantly higher in patients with DR compared to those without DR, 32.72 (IQR 32.52) vs 21.37 (IQR 14.93) ng/mL, respectively (*p* < 0.05). However, tear PAI-1 were comparable in both groups. Serum and tear tPA levels in both groups were also comparable (*p* > 0.05). Among patients with DR, there were no significant correlations between tear and serum of both biomarkers. Patients without DR showed a moderate positive correlation between serum and tear tPA levels with a coefficient of 0.363, albeit no statistical significance. Patients with DR demonstrated a significant positive correlation between levels of tears PAI-1 and BMI (r = 0.555, *p* = 0.026). In the group without DR, there was a statistically significant positive correlation between serum level of PAI-1 with urine albumin creatinine ratio (UACR) (r = 0.501, *p* = 0.013).

**Conclusion:**

The present study demonstrated a significantly greater serum PAI-1 levels in patients with DR compared to those without DR. No significant correlations between tears and serum PAI-1 and tPA were observed. Thus, the role of tear biomarkers remains relevant for further investigations.

## Introduction

Type 2 diabetes mellitus (T2DM) is an emerging global epidemic and a major concern for public health. It is associated with many debilitating macro- and micro-vascular complications. According to the International Diabetes Federation (IDF) 9^th^ Edition in 2019, 463 million people have diabetes worldwide, out of which 163 million were in the Western Pacific Region. A modest estimation indicates that this figure will increase to 212 million by 2045 [1]. In Malaysia, the National Health Morbidity Surveys (NHMS) has reported a steady rise in the prevalence of T2DM from 8.3% in 1996, to 13.4% in 2015, and to 18.3% according to the last survey in 2019, which could be translated to almost 2 million adults ≥ 18 years old [2].

Diabetic retinopathy (DR) is a serious complication of longstanding and poorly controlled T2DM that could range from mild disease to severe proliferative retinopathy, the forerunner in causing blindness and visual disability [3]. In order to facilitate screening and early detection of the disease, studies have been done to evaluate usability of tear proteins as biomarkers. Kim et al. reported that the tear levels of lipocalin-1, heat-shock protein 27 and β2-macroglobulin progressively reduce in patients with DR [4]. One of the studies identified lipocalin 1, lacritin, lysozyme C, lactotransferrin, lipophilin A and immunoglobulin lambda chain as possible biomarker candidates as the levels of these proteins were significantly greater in the tear of patients with DR [5]. Elevated level of TNF-alpha, an inflammatory cytokine, was detected in tears of DR patients and a positive correlation was observed with the severity of DR [6]. In another study, the data from tear fluid proteomics analysis and digital microaneurysm detection on fundus images were integrated as the input of a machine learning system.It was concluded that the combined model can be considered a reliable screening method [7].

Tissue plasminogen activator (t-PA) and plasminogen-activator inhibitor-1 (PAI-1) have also been detected in tears. PAI-1 is a serine protease inhibitor primarily secreted by vascular endothelial cells, hepatocytes and adipocytes. PAI-1 is the primary inhibitor of tPA and acts as main negative regulator of fibrinolysis process [8]. PAI-1 has been shown to be highly expressed in the presence of macrovascular disorders in subjects with DM [9]. Notably, studies have also suggested association of increased PAI-1 expression with microvascular complications of DM such as nephropathy and retinopathy [10]. However, Polat et al. reported no significant difference in serum PAI-1 levels among patients with type I diabetes with or without retinopathy [11]. tPA is a serine protease that cleaves plasminogen to plasmin. It plays a key role in the fibrinolytic system. Vascular endothelium is the major source of tPA in circulation. Lower levels of circulating tPA have been detected in diabetic subjects. It has been reported that fibrinolytic activity is reduced in patients with long-standing diabetes which in turn promotes the associated microthromboembolism. In fact a causal relationship between reduced tPA and microangiopathy resulting in characteristic retinal ischemia in diabetic retinopathy was proposed by *Little* in 1981 [12]. Further studies revealed a significantly higher level of plasma PAI-1 in DM subjects with DR when compared to subjects without DR [13]. According to the VADT study in patients with T2DM, higher serum PAI-1 levels are associated with increased future risks of DR [14]. However, it is still unknown if there is a correlation between plasma and tears PAI-1 and tPA levels in patients with DR. Hence, this study aimed to investigate the correlation of tears and serum levels of PAI-1 and tPA with DR. Current study also determined the correlations of serum PAI-1 and tPA levels with their tear levels in patients with and without DR.

## Material and methods

This was a cross-sectional study involving T2DM patients with or without DR receiving treatment at the Endocrine Clinic of the Universiti Teknologi MARA. The study was approved by Universiti Teknologi MARA Research Ethics Committee (REF:600-IRMI (5/1/6). The sample size for this study was calculated using the Open-EPI software for comparison using 2 mean formula to achieve 95% confidence interval and study power of 80% using two mean comparison in reference to results reported by Takada et al. [15] Therefore, total sample size (n) needed was 52. After including 15% attrition the total sample size was 60.

Subjects were recruited using convenient sampling, with inclusion criteria as follows: Age 18 years and above; established diagnosis of T2DM; disease duration more than ≥ 5 years; HbA1c ≤ 12; stable doses of anti-diabetic medications for the past 3 months and estimated glomerular filtration rate of more than 60 ml/min/1.73m^2^. Patients were excluded if they had the following: uncontrolled blood pressure > 160/90 mmHg; type 1 diabetes mellitus; eye surgery during the past 3 months; glaucoma on treatment; cataract involving posterior subcapsular cataract with vision less than 6/12 and nucleus sclerosis grade III and above); evidence of acute conditions or recent hospitalization for ongoing infection, surgery or any other condition resulting in fluctuations of glycaemic control; severe dryness with any inflammatory conditions of the eye; patients on allopurinol or colchicine and those receiving treatment for ischaemic heart disease. In order to rule out ophthalmic pathologies, all subjects underwent best-corrected visual acuity, tonometry, anterior segment slit lamp biomicroscopy and Schirmer test. Severe dryness is defined as a measurement of less than 5 mm on the filter paper during the Schirmer test. Coloured fundus photographs were taken from the subjects by digital fundud camera (CR-2 AF, Canon Inc, Japan) and were examined according to the Early Treatment Diabetic Retinopathy Study (ETDRS) standards. Based on the outcome of the investigation, subjects were grouped into two categories. Group I was defined as those without DR and Group II with DR.

Patients who fulfilled the inclusion and exclusion criteria were invited to participate and written informed consent was obtained from all subjects. Once the subjects agreed to participate in the study, they were required to attend the clinic once for collection of tears from both eye and, blood and urine samples. The samples were collected in the morning between 8–10 am to avoid diurnal variations in the levels of target proteins [16, 17].

For collecting tear samples, the Schirmer tear test (STT) strips were used as described previously [6, 18]. Accordingly, the tip of the strip was placed under the lower eyelid in the inferior conjunctival fornix and participants were asked to keep the eyes closed for 5 min to allow absorption of tears. Care was taken not to stimulate tear secretion during the procedure. For extraction of tear fluid from STT, the strip was placed in a 0.5 ml tube that was punctured at the bottom with a cannula and was placed in a larger 1.5 ml tube. This was followed by centrifugation at 16000xg at 4ºC for 5 min. The tear fluid is pulled out from the Schirmer strip by the centrifugal force, through central “pore” of the punctured smaller tube and flows into the 1.5 ml outer tube. The samples were stored at -80 ºC till further processing [18].

The venous blood was collected in a volume of 10 ml after a minimum 8-h fasting. The plasma was separated by centrifugation for 15 min at 1000xg at 2–8 ˚C and kept at -80˚C until further analysis, which was conducted in Institute of Medical Molecular Biotechnology, Universiti Teknologi MARA. In addition to the levels of tPA and PAI-1, blood samples were also examined for fasting blood glucose (FBG), urea, creatinine, fasting lipid profile (total cholesterol, low-density lipoprotein cholesterol, triglycerides, high-density lipoprotein cholesterol), C-reactive protein (CRP) and uric acid. Biochemical measurements were performed according to standard laboratory procedures. FBG was analyzed using the hexokinase method, high-performance liquid chromatography for HbA1c while total cholesterol (TC), triglyceride (TG) and high-density lipoprotein-cholesterol (HDL-c) and uric acid were measured by enzymatic reference methods. Low-density lipoprotein-cholesterol (LDL-c) was derived using Friedwald equation. CRP was measured by using particle enhanced turbidimetric assay. The spot urine samples were analysed for urine microalbumin following the laboratory protocol and urine albumin-creatinine-ratio (UACR) was calculated as albumin concentration divided by creatinine concentration. Serum extracted from whole blood and tear samples were analyzed for PAI-1 and tPA concentrations by enzyme-linked immunosorbent assay (ELISA). The levels of tPA and PAI-1 in tears and plasma were measured using human tPA ELISA Kit (Catalog No: E-EL-H2106 96 T) and human PAI-1 ELISA Kit (Catalog No: E-EL-H2104 96 T), respectively, according to the manufacturer’s instructions (Elabscience Biotech, China). Briefly, for the estimation of both the tPA and PAI-1, 100μL standard or sample was pipetted into the designated wells and incubated for 90 min at 37 °C. Then, 100 μL of biotinylated detection antibody working solution was added to each well and incubation was done for 60 min at 37 °C. Subsequently, the plate was washed 3 times and 100 μL of HRP conjugate working solution was added. After incubation for 30 min at 37 °C, plate was washed again and 90 μL of substrate reagent was added. After further incubation for 15 min at 37 °C, 50 μL of stop solution was added. The absorbance was read immediately at 450 nm using a microplate reader (Victor™ X5, Perkin Elmer).

### Statistical analysis

SPSS Windows Version 25.0 (Statistical Package for the Social Sciences, SPSS Inc., Chicago, IL, USA) was used to analyze the data collected. The missing data were listwise deleted. Non-parametric data are presented as median (interquartile range) and parametric data as mean ± SD. P-values are results of Mann–Whitney test for continuous data and Fisher's exact test for categorical data.. Chi-squared and Fisher’s exact test were used to assess the differences between the categorical variables. Unpaired* t* test was used for calculating significant differences between continuous variables.

Group with DR and without DR were compared using Chi-Square tests for categorical variables and independent-t test for continuous variables. A *p* value < 0.05 is considered significant. Among those with DR, 13 had mild disease, 11 had moderate and only 3 had severe retinopathy. Thus, we were not able to further categorise the sample based on severity of DR. Correlation between two continuous variables were analysed using Spearmans rho test for non-parametric data. Cohen’s (1988) cut-off points were used for interpretation of the strength of correlation. Accordingly, a value of r ≥ 0.5 showed a strong correlation whereas 0.3 – 0.5 showed a moderate correlation. Weak and insubstantial correlation was indicated by r < 0.3 – 0.1 and < 0.1, respectively.

## Results

A total of 55 patients (32 females and 23 males; mean age 52.37 ± 10.8 years; range 32—72 years) fulfilled the inclusion and exclusion criteria and consented to participate in the study. The subject characteristics are presented in Table 1.

**Table 1:** Comparison of the sociodemographic and baseline characteristics of study population

Variables	T2DM patients			
	Total subjects *n* = 55 (%)	DR *n* = 26 (%)	No DR *n* = 29 (%)	*p* value
Age, (Years, mean ± SD),	52.37 ± 10.80	52.16 ± 8.54	52.55 ± 12.63	0.894
Ethicity Malay, *n (*%) Non-malay, *n (*%)	43 (78.2) 12 (21.8)	21 (80.8) 5 (19.2)	22 (75.9) 7 (24.1)	0.660
Gender Male *n (*%) Female *n (*%)	23 (41.8) 32 (58.2)	9 (34.6) 17 (65.4)	14 (48.3) 15 (51.7)	0.305
**DM durations, (Years, median (IQR))**	**11.00 (8)**	14.50 (10)*	**9.00 (6)**	**0.017**
Smoking history: Smoked, *n (*%) Never smoked, *n (*%)	8 (14.5) 47 (85.5)	2 (7.7) 24 (92.3)	6 (20.7) 23 (79.3)	0.257
Family history of DM: Yes, *n (*%) No, *n (*%)	53 (96.4) 2 (3.6)	25 (96.2) 1 (3.8)	28 (96.6) 1 (3.4)	1.000
Hypertension Yes, *n (*%) No, *n (*%)	36 (65.5) 19 (34.5)	18 (69.2) 8 (30.8)	18 (62.1) 11 (37.9)	0.577
BMI (Kg/m2, mean ± SD)	30.69 ± 5.65	30.70 ± 5.87	30.68 ± 5.55	0.987
Asian BMI, *n (*%) ≤ 22.9 (non-obese) 23.0 – 27.4 (pre-obese/overweight) ≥ 27.5 (obese)	4 (7.3) 15 (27.3) 36 (65.5)	3 (11.5) 5 (19.2) 18 (69.2)	1 (3.4) 10 (34.5) 18 (62.1)	0.302
**SBP, median (IQR), mmHg**	**140.00 (20.00)**	145.00 (14.06)*	**132.00 (23.00)**	**0.022**
DBP, (mean ± SD), mmHg	76.84 ± 7.77	74.96 ± 9.03	78.52 ± 6.11	0.098
Hba1c (%), median (IQR)	7.80 (1.8)	8.20 (2.5)	7.40 (1.8)	0.104
UACR, median (IQR), mg/mmol	2.20 (10.7)	1.90 (6.3)	5.30 (13.9)	0.100
UACR Level (mg/mmol), *n (*%) < 3 3–29 ≥ 30	30 (54.5) 20 (36.4) 5 (9.1)	12 (40.0) 11 (55.0) 3 (60.0)	18 (62.1) 9 (31.0) 2 (6.9)	0.558
FBS, median (IQR), mmol/L	6.90 (3.1)	6.60 (4.0)	7.40 (3.1)	0.886
Creatinine, mean ± SD,µmol/L	72.93 ± 16.51	71.70 ± 13.76	74.03 ± 18.80	0.606
Uric acid, median (IQR),µmol/L	326.00 (123.00)	318.00 (122.50)	335.00 (150.00)	0.886
Total cholesterol, median (IQR), mmol/L	3.90 (1.4)	3.85 (1.3)	4.00 (1.9)	0.933
Triglycerides, median (IQR), mmol/L	1.50 (0.6)	1.50 (1.0)	1.50 (0.4)	0.483
HDL-cholesterol, median (IQR), mmol/L	1.20 (2.8)	1.30 (0.4)	1.10 (0.6)	0.728
LDL-cholesterol, median (IQR), mmol/L	2.00 (0.6)	1.95 (0.9)	2.00 (1.4)	0.755
CRP,median (IQR), mg/L	1.80 (3.6)	1.85 (4.1)	1.80 (3.7)	0.787
eGFR, mean ± SD, ml/min/1.73 m2	91.41 ± 18.18	91.12 ± 16.63	91.68 ± 19.75	0.910

Non-parametric data are presented as median (interquartile range) and parametric data as mean ± SD. *P*-values are results of Mann–Whitney test for continuous data and Fisher's exact test for categorical data.. *P*-values are results of Mann–Whitney test for continuous data for between the groups, and Fisher's exact test for categorical data. *BMI* Body mass index, *DBP* Diastolic blood pressure, *HbA1c* Hemoglobin A1c, *FBS* Fasting blood sugar, *SBP* Systolic blood pressure, *UACR* Urinary albumin-to-creatinine ratio, *HDL-C* High-density lipoprotein, *LDL-C* Low-density lipoprotein, *CRP* C-reactive protein, *eGFR* estimated glomerular filtration rate. *P* value < 0.05 is considered significant. *NS* Not significant

Comparison between the patients with DR and without DR revealed a significantly longer disease duration of 14.5 (IQR 10) years among those with DR compared to 9 (IQR 6) years among those without DR (p = 0.017). Similarly, the SBP of 145 (IQR 14) mmHg was significantly higher in the group with DR compared to 132 (IQR 23) mmHg in the group without DR (p = 0.022). (Table 1).

Serum PAI-1 level was significantly greater in patients with DR compared to those without DR, with a median value of 32.72 (IQR 32.52) vs 21.37 (IQR 14.93) ng/mL, respectively. The difference amounted to 1.53 folds (*p* < 0.05). However, the tear PAI-1 levels in patients with and without DR were comparable (*p* > 0.05) (Table 2).

**Table 2:** Serum and tear PAI-1 and tPA levels among subjects with Type 2 Diabetes with or without retinopathy

Parameters	Median (IQR)		*P* value*
	DR	No DR	
PAI-1 level in tears (ng/ml),	0.24 (0.06)	0.22 (0.03)	0.122
PAI-1 level in blood (ng/ml),	32.72 (32.52)	21.37 (14.93)	0.025**
tPA level in tears (ng/ml)	0.46 (0.02)	0.46 (0.12)	0.582
tPA level in blood (ng/ml)	3.48 (5.06)	3.94 (6.72)	0.090

Data are presented as median (interquartile range). *PAI-1* Plasminogen activator inhibitor-1, *tPA* tissue plasminogen activator

^**^P value < 0.05 (Mann–Whitney test)

Serum tPA level although showed a lower median value of 3.48 (IQR 5.06) ng/ml in DR group compared to 3.94 (IQR 6.72) ng/ml in the group without DR, the difference did not reach the significant level (*p* < 0.09). The tear tPA levels did not show significant difference with median values of 0.46 (0.02) ng/ml and 0.46 (0.12) ng/ml, respectively. Notably, in half of the patients (13 out of 26) with DR the tear tPA levels were not detectable; however in patients without DR the tear tPA level were not detectable in 8 (out of 29) patients (Table 2).

Among the patients with DR (*n* = 26), 14 patients had mild DR and 13 patients had moderate to severe. The average of serum PAI-1 level among the patients with mild DR was 37.34 ng/mL, while moderate to severe was 28.95 ng/mL. Tears PAI-1 levels were similar between those with mild and moderate to severe DR at 0.21 ng/mL. Serum tPA levels were 4.03 ng/ml and 6.06 ng/ml in mild and moderate to severe DR, respectively. Tear tPA level was 0.53 ng/mL in those with mild DR and 0.58 ng/mL in those with moderate to severe DR.

We performed univariate analyses to determine associations between the serum and tear levels of two proteins. Among patients with DR no correlation was noted between serum and tear PAI-1 levels with a Spearman’s coefficient of 0.028 (*p* = 0.928). In this group, there was also no correlation between serum and tear tPA levels. Among patients without DR, no significant correlation was observed between serum and tear PAI-1; however, the serum and tear tPA levels showed a positive and moderate correlation with a coefficient of 0.363 although the p value was not significant.

We also measured correlation of the serum and tear levels of two proteins with other variables. Notably, there was a significant positive correlation between tears level of PAI-1 with BMI (r = 0.555, *p* = 0.026) in DR group. In the group without DR, there was a statistically significant positive correlation between plasma level of PAI-1 with UACR (r = 0.501, *p* = 0.013). We did not observe a correlation between PAI-1 and tPA levels in serum and tears with any other variable in both the groups with and without DR. (Table 3 and 4).

**Table 3:** Univariate analysis demonstrating correlation of serum and tear PAI-1 levels with other variables in patients with DR

Parameters	PAI-1 tears	PAI-1 blood	tPA tears	tPA blood
	r^a^ (*p* value)^b^	r (*p* value)	r (*p* value)	r (*p* value)
BMI	0.555 (0.026)*	0.194 (0.388)	-0.271 (0.449)	0.427 (0.060)
Age	-0.099 (0.715)	0.230 (0.304)	-0.105 (0.774)	0.024 (0.920)
DM duration	-0.010 (0.972)	0.183 (0.416)	0.031 (0.933)	0.175 (0.459)
HbA1c	0.377 (0.150)	-0.219 (0.327)	0.240 (0.504)	-0.005 (0.982)
FBS	0.120 (0.659)	-0.267 (0.229)	0.412 (0.236)	-0.009 (0.970)
CRP	0.289 (0.278)	0.281 (0.205)	0.086 (0.813)	0.255 (0.278)
Uric acid	0.325 (0.219)	-0.119 (0.597)	-0.394 (0.260)	0.146 (0.539)
UACR	-0.075 (0.782)	0.056 (0.085)	0.080 (0.826)	-0.107 (0.654)

^a^Spearman correlation test

^b^*P* value < 0.05

^*^correlation is significant at the 0.05 level

*BMI* Body mass index, *HbA1c* Hemoglobin A1c, *DM* Diabetes mellitus, *FBS* Fasting blood sugar, *CRP* C-reactive protein, *UACR* Urinary albumin-to-creatinine ratio

**Table 4:** Univariate analysis demonstrating correlations of serum and tear PAI-1 levels with other variables in diabetic patients without retinopathy

Parameters	PAI-1 tears	PAI-1 blood	tPA tears	tPA blood
	r ª(*p* value)^b^	r (*p* value)	r (*p* value)	r (*p* value)
BMI	0.317 (0.131)	0.161 (0.552)	0.166 (0.486)	0.309 (0.213)
Age	-0.009 (0.974)	0.208 (0.330)	-0.330 (0.155)	-0.249 (0.320)
DM duration	0.071 (0.793)	-0.026 (0.903)	0.114 (0.633)	0.384 (0.116)
HbA1c	0.040 (0.884)	-0.021 (0.921)	-0.011 (0.962)	0.081 (0.751)
FBS	-0.015 (0.957)	-0.090 (0.676)	-0.265 (0.260)	-0.152 (0.548)
CRP	-0.344 (0.191)	0.360 (0.084)	-0.035 (0.883)	0.242 (0.334)
Uric acid	0.117 (0.665)	0.034 (0.875)	-0.049 (0.838)	-0.203 (0.418)
UACR	-0.187 (0.489)	**0.501 (0.013)**	-0.035 (0.883)	0.041 (0.870)

^a^Spearman correlation test

*P* value < 0.05

*BMI* Body mass index, *HbA1c* Hemoglobin A1c, *DM* Diabetes mellitus, *FBS* Fasting blood sugar, *CRP* C-reactive protein*, UACR* Urinary albumin-to-creatinine ratio

All the variables with *p* < 0.25 from the univariate analyses were further analysed with multivariable regression analysis. There were no statistically significant findings including duration of DM as well as systolic and diastolic blood pressures.

## Discussion

This study was carried out to determine the association of serum and tear levels of tPA and PAI-1 among patients with and without DR irrespective of the stage of DR. It was observed that serum PAI-1 level was significantly greater in patients with DR compared to those without DR, however, the tear PAI-1 levels in patients with and without DR were comparable. Similar observations were not made for tPA levels. We did not observe a correlation between tear PAI-1 and serum PAI-1 levels. Similarly, we did not demonstrate correlation between tear and serum tPA level.

As one of the common complications of diabetes, DR requires an early intervention and treatment to prevent disease progression and blindness. An easy detection of biomarkers in body fluids that are easily accessible may be of great importance for effective disease management. Since, high PAI-1 and low tPA levels are known to be associated with DR, current study was carried out to determine if these proteins can be detected in tears and if their tear levels correlate with their corresponding serum levels and other disease related parameters.

T2DM is a well-known predisposing factor for atherosclerotic coronary artery disease development and precipitation of myocardial infarction. Hence, over the past few decades significant interest has been generated on the effects of diabetes on fibrinolytic system in blood which involves plasminogen activators, particularly t-PA and its inhibitor PAI-1. t-PA is a serine protease and it catalyzes conversion of circulating plasminogen to plasmin. Plasmin is a relatively non-specific protease that breaks fibrin into its degradation products. Since, both plasminogen and t-PA bind specifically to clot associated fibrin, the action of t-PA is largely clot-specific and helps to lyse thrombi [19]. PAI-1, on the other hand, inhibits t-PA associated with clots; hence inhibiting its ability to lyse nascent thrombi. Besides the association of increased levels of PAI-1 with coronary artery disease in patients with diabetes, higher level of PAI-1 at baseline was reported to be an independent risk factor for the onset of DR. In this study 858 patients were followed up over 5 years [14]. In another study, elevated serum PAI-1 levels were significantly associated with end-stage proliferative DR [20]. Since an association of elevated serum PAI-1 levels with development of DR has been reported by many researchers, we considered estimating its level in tears of patients with DR and compare with those without DR. In accordance with earlier reports, we observed significantly elevated levels of serum PAI-1 in patients with DR compared to those without DR (*p* < 0.05). However, no significant difference was observed for the tear level of PAI-1 between the two groups. Serum tPA level although showed a lower value among patients with DR, the differences from those without DR did not reach a significant level (*p* = 0.09). Additionally, we did not observe a significant difference in the tear levels of tPA between the two groups of patients. Notably, in half of the patients with DR the tear level of tPA were not detectable indicating its very low expression in tears. However, a moderate correlation was observed between serum and tear tPA levels among patients without DR. A similar observation was not made for PAI-1 levels. It is likely that a local regulatory mechanism influences the tear level of PAI-1 and hence the lack of correlation. However, considering that the number patients included in the current study was rather small, further investigations involving larger cohort of patients may be useful for confirming the association of level of these two proteins in tears with those in serum.

In this study we also estimated the correlation of serum and tear levels of PAI-1 and tPA with other variables. Notably, a significant association was observed for tear PAI-1 levels with BMI among patients with DR. In a previous study, increased PAI-1 activity was found to be associated with an increased waist circumference among diabetic subjects [21]. Other studies have also indicated that serum PAI-1 levels are positively correlated with obesity and insulin resistance [22–24]. The current study, for the first time observed a significant association of tear PAI-1 level with BMI among patients with DR. Although, previous studies have reported as association of serum PAI-1 with obesity and insulin resistance, in the current study we did not observe this association [25]. A strong link between inhibition of fibrinolysis in obese patients and the elevation of PAI-1 has been described [26] and a decrease in BMI among individuals with morbid obesity was shown to cause a favorable effect on the fibrinolytic system due to a decrease in PAI-1 levels [27]. It is likely that the association is prominently present in obese individuals and is not significant in those with BMI not amounting to obesity as was the case in the current study.

A positive association was also found between serum PAI-1 level and UACR among patients without DR in this study. In accordance with our observation, one of the previous studies has shown that serum levels of PAI-1 are significantly increased in diabetic patients with higher urine albumin excretion [28]. In another study, inhibition of PAI-1 prevented kidney injury in diabetic mice. Accordingly, the association of serum PAI-1 with UACR level was observed in the current study. However, the same was not evident among patients with DR. Although, the reason for this remains unclear, it is likely that the gender differences (M:F of 9:17 in the DR group compared to 14:15 in no DR group) were the contributing factors. One of the previous studies has suggested that factors that correlate with albuminuria were different for men and women according to comorbidities such as hypertension and diabetes [29]. Additionally, other factors that may have contributed include the severity of DR and nephropathy, ongoing medications as well as the glycaemic control, although a positive correlation with HbA1c level was not evident in the current study. We also did not observe any correlation between PAI-1 and tPA levels with any other variable.

In conclusion, this current study detected the presence of PAI-1 and tPA in the tears of diabetic patients with and without DR. The serum PAI-1 levels were significantly greater in patients with DR compared to those without DR. A positive correlation was observed between tear and serum tPA levels in patients without DR. Perhaps inclusion of a larger cohort of patients may reveal the potential of PAI-1 and tPA to be used as potential biomarkers in the management of DR. Further studies involving healthy controls could also provide a better comparative data, which was a limitation of the current study.

## Data Availability

The datasets used and analysed during the current study available from the corresponding author on reasonable request.

## Abbreviations

DR: Diabetic retinopathy
T2DM: Type 2 diabetes mellitus
IQR: Interquartile range
UACR: Urine albumin-to-creatinine ratio
PAI-1: Plasminogen activator inhibitor-1
tPA: Tissue plasminogen activator
BMI: Body mass index
CRP: C-reactive protein
HbA1c: Hemoglobin A1c
FBG: Fasting blood glucose

## References

[1] International Diabetes Federation. IDF Diabetes Atlas. 9th ed. Brussels, Belgium: International Diabetes Federation. 2019.

[2] Institute for Public Health (IPH), National Institutes of Health, Ministry of Health Malaysia. National Health and Morbidity Survey (NHMS) 2019: Vol. I: NCDs – Non-Communicable Diseases: Risk Factors and other Health Problems. 2020.

[3] Klein BEK. Overview of Epidemiologic Studies of Diabetic Retinopathy. Ophthalmic Epidemiol. 2007;14(4):179–83. 10.1080/09286580701396720 17896294

[4] Kim HJ, Kim PK, Yoo HS, Kim CW. Comparison of tear proteins between healthy and early diabetic retinopathy patients. Clin Biochem. 2012;45(1–2):60–67. 10.1016/j.clinbiochem.2011.10.006 22040812

[5] Csősz É, Boross P, Csutak A, Berta A, Tóth F, Póliska S, Török Z, Tőzsér J (2012). Quantitative analysis of proteins in the tear fluid of patients with diabetic retinopathy. J Proteomics.

[6] Costagliola C (2013). TNF-alpha levels in tears: a novel biomarker to assess the degree of diabetic retinopathy. Mediators Inflamm.

[7] Torok Z, Peto T, Csosz E, Tukacs E, Molnar AM, Berta A, Tozser J, Hajdu A, Nagy V, Domokos B, Csutak A, Combined Methods for Diabetic Retinopathy Screening, Using Retina Photographs and Tear Fluid Proteomics Biomarkers. Diabetes Res. 2015;2015:623619. 10.1155/2015/623619 PMC4499636 26221613

[8] Serdiuk VN, Kyryliuk ML, Pylypenko LYu. Activity of plasminogen activator inhibitor-1 in blood of patients with metabolic syndrome depending on a stage of diabetic retinopathy. J Ophthalmol (Ukraine). 2018;3:52–56.

[9] Lyon CJ, Hsueh WA (2003). Effect of plasminogen activator inhibitor-1 in diabetes mellitus and cardiovascular disease. Am J Med.

[10] Altalhi R, Pechlivani N, Ajjan RA (2021). PAI-1 in Diabetes: Pathophysiology and Role as a Therapeutic Target. Int J Mol Sci.

[11] Polat SB, Ugurlu N, Yulek F, Simavli H, Ersoy R, Cakir B, Erel OJ (2014). Evaluation of serum fibrinogen, plasminogen, α2-anti-plasmin, and plasminogen activator inhibitor levels (PAI) and their correlation with presence of retinopathy in patients with type 1 DM. Diabetes Res.

[12] Little HL. Alteration in blood elements in the pathogenesis of diabetic retinopathy. Ophthalmology. 1981;88(7):647–54. 10.1016/s0161-6420(81)34971-9 7267033

[13] Erem C (2005). Coagulation and fibrinolysis parameters in type 2 diabetic patients with and without diabetic vascular complications. Med Princ Pract.

[14] Azad N (2014). Association of PAI-1 and fibrinogen with diabetic retinopathy in the Veterans Affairs Diabetes Trial (VADT). Diabetes Care.

[15] Takada Y (1993). Changes in fibrinolytic parameters in male patients with type 2 (non-insulin-dependent) diabetes mellitus. Thromb Res.

[16] Andreotti F, Kluft C (1991). Circadian variation of fibrinolytic activity in blood. Chronobiol Int.

[17] Angleton P, Chandler WL, Schmer G (1989). Diurnal variation of tissue-type plasminogen activator and its rapid inhibitor (PAI-1). Circulation.

[18] Posa A (2013). Schirmer strip vs. capillary tube method: non-invasive methods of obtaining proteins from tear fluid. Ann Anat.

[19] Van de Werf F (1984). Coronary thrombolysis with tissue-type plasminogen activator in patients with evolving myocardial infarction. N Engl J Med.

[20] Zhong ZL, Chen S (2012). Plasma plasminogen activator inhibitor-1 is associated with end-stage proliferative diabetic retinopathy in the Northern Chinese Han population. Exp Diabetes Res.

[21] Al-Hamodi Z (2011). Association of plasminogen activator inhibitor-1 and tissue plasminogen activator with type 2 diabetes and metabolic syndrome in Malaysian subjects. Cardiovasc Diabetol.

[22] Juhan-Vague I (2003). Plasminogen activator inhibitor-1, inflammation, obesity, insulin resistance and vascular risk. J Thromb Haemost.

[23] Loskutoff DJ, Samad F (1998). The Adipocyte and Hemostatic Balance in Obesity. Arterioscler Thromb Vasc Biol.

[24] Ma LJ (2004). Prevention of obesity and insulin resistance in mice lacking plasminogen activator inhibitor 1. Diabetes.

[25] Schneider DJ, Sobel BE (2012). PAI-1 and Diabetes: A Journey From the Bench to the Bedside. Diabetes Care.

[26] Mutch NJ, Wilson HM, Booth NA (2001). Plasminogen activator inhibitor-1 and haemostasis in obesity. Proc Nutr Soc.

[27] Solá E, Vayá A, España F, Castelló R, Ramón LA, Hernández-Mijares A, Vicente V, Estellés A. Plasminogen activator inhibitor-1 levels in severe and morbid obesity. Effect of weight loss and influence of 4G/5G polymorphism. Thromb Res. 2008;122(3):320–7. 10.1016/j.thromres.2007.10.016 18037477

[28] Zakariaa E, Hossam Al-Din M, Ghanem NS, Sadik NA, Assem M, Taha F. Urinary albumin excretion and progression of renal disease with impaired fibrinolytic activity in type 2 diabetes mellitus. The Egyptian J Int Med. 2015;27:08–114.

[29] Jang M, Oh S, Noh HM, Chun S, Oh HY, Park KH, Paek YJ, Song HJ (2015). Differences in Factors Associated with Albuminuria according to Gender and Comorbidities of Hypertension and Diabetes. Korean J Fam Med.

